# Recent Advances in Two-Dimensional Magnets: Physics and Devices towards Spintronic Applications

**DOI:** 10.34133/2020/1768918

**Published:** 2020-06-19

**Authors:** Vertikasari P. Ningrum, Bowen Liu, Wei Wang, Yao Yin, Yi Cao, Chenyang Zha, Hongguang Xie, Xiaohong Jiang, Yan Sun, Sichen Qin, Xiaolong Chen, Tianshi Qin, Chao Zhu, Lin Wang, Wei Huang

**Affiliations:** ^1^Key Laboratory of Flexible Electronics (KLOFE) & Institute of Advanced Materials (IAM), Nanjing Tech University (Nanjing Tech), 30 South Puzhu Road, Nanjing 211816, China; ^2^Frontiers Science Center for Flexible Electronics (FSCFE), Shaanxi Institute of Flexible Electronics (SIFE) & Shaanxi Institute of Biomedical Materials and Engineering (SIBME), Northwestern Polytechnical University (NPU), 127 West Youyi Road, Xi'an 710072, China; ^3^Department of Electrical and Electronic Engineering, Southern University of Science and Technology, Shenzhen 518055, China; ^4^Center for Programmable Materials, School of Materials Science and Engineering, Nanyang Technological University, Singapore 639798, Singapore

## Abstract

The emergence of low-dimensional nanomaterials has brought revolutionized development of magnetism, as the size effect can significantly influence the spin arrangement. Since the first demonstration of truly two-dimensional magnetic materials (2DMMs) in 2017, a wide variety of magnetic phases and associated properties have been exhibited in these 2DMMs, which offer a new opportunity to manipulate the spin-based devices efficiently in the future. Herein, we focus on the recent progress of 2DMMs and heterostructures in the aspects of their structural characteristics, physical properties, and spintronic applications. Firstly, the microscopy characterization of the spatial arrangement of spins in 2D lattices is reviewed. Afterwards, the optical probes in the light-matter-spin interactions at the 2D scale are discussed. Then, particularly, we systematically summarize the recent work on the electronic and spintronic devices of 2DMMs. In the section of electronic properties, we raise several exciting phenomena in 2DMMs, i.e., long-distance magnon transport, field-effect transistors, varying magnetoresistance behavior, and (quantum) anomalous Hall effect. In the section of spintronic applications, we highlight spintronic devices based on 2DMMs, e.g., spin valves, spin-orbit torque, spin field-effect transistors, spin tunneling field-effect transistors, and spin-filter magnetic tunnel junctions. At last, we also provide our perspectives on the current challenges and future expectations in this field, which may be a helpful guide for theorists and experimentalists who are exploring the optical, electronic, and spintronic properties of 2DMMs.

## 1. Introduction

Magnetism has always been a classical and important subject for academic studies and application devices, to which low dimensionalities give new physical significance due to the strong quantum confinement effect. In the past decades, a rich variety of nanoscale magnetic materials have long been pursued by scientists [1–3]. In particular, two-dimensional magnetic materials (2DMMs) have attracted enormous attention, owing to their merits for the simple integration of multiple-layered heterostructures and the full tunability by external electric fields [4]. At first, researchers have realized and investigated 2D magnetism in fabricating various magnetic films, such as transition metal oxides and magnetic alloys [5]. From that, many interesting physical phenomena and device configurations have emerged [6]. For instance, *d*-zero ferromagnetism can be introduced into nonmagnetic films through strong interface coupling with magnetic materials [5, 7]. However, there remain a lot of challenges for these magnetic films with quasi-2D morphology: (1) the absence of intrinsic 2D crystal structure; (2) the structural instability at the truly 2D scale; (3) the vanishing of magnetic order in the ultrathin limit; and (4) the requirement of good lattice matching with substrates and adjacent layers, and so forth. Given that, it is highly desirable to discover and develop truly 2DMMs for novel magnetic properties and high-compacted devices.

**Figure 1:**
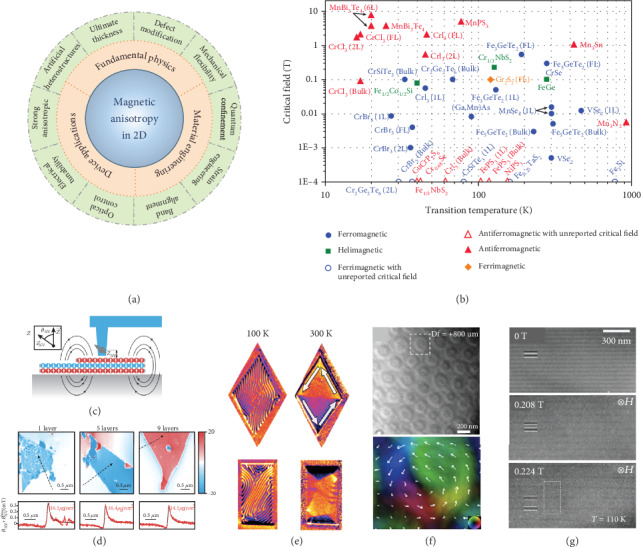
(a) Attributes of 2D magnetic materials. (b) Plotting of transition temperatures and critical fields of representative 2D magnetic materials. (c) Scheme of the working principle and setup of scanning single-spin magnetometry technique. (d) Magnetization maps of monolayer, pentalayer, and nonalayer of CrI_3_ at 7 K. Lower row: the data of stray magnetic field *B*_NV_ measured across the edges of each flake, along the lines indicated in the maps. (e) Magnetic domain mapping of 250 nm Fe_3_GeTe_2_ microstructures (diamond and rectangular shape) at 110 K and 300 K, respectively. The data at 110 K exhibits stripe-like feature in the out-of-plane magnetization component for both these two microstructures, while at 300 K, in-plane magnetization component plays a dominant role: a magnetic vortex state in the diamond-shaped microstructure and a multidomain state in the rectangular-shaped microstructure. (f) Overfocused Lorentz TEM images of the skyrmion bubbles of Fe_3_GeTe_2_ at 93 K and zero field (upper panel) and a zoom-in image of the in-plane magnetization distribution map for the skyrmion bubble outlined by a white dashed box (bottom panel). (g) Underfocused Lorentz TEM images of 70 nm Cr_1/3_NbS_2_ evolving with in-plane magnetic field at 110 K. Panels (c, d) are reproduced with permission from ref. [16], copyright 2019 *Science*. Panel (e) is reproduced with permission from ref. [17], copyright 2018 *Nano Letters*. Panel (f) is reproduced with permission from ref. [21], copyright 2019 *Nano Letters*. Panel (g) is reproduced with permission from ref. [22], copyright 2012 *Physical Review B*.

Since the discovery of transport properties in graphene [8, 9], new physical phenomena of 2D materials are being continuously revealed in a wide range of fields [10–12]. As for magnetism, intrinsic magnetic order in the monolayer/few-layer limit was firstly experimentally realized in 2D ferromagnets Cr_2_Ge_2_Te_6_ and CrI_3_ in 2017, after which various 2DMMs have been rapidly discovered and studied [13, 14]. In contrast to the conventional magnetic materials, the magnetic order of 2DMMs can persist down to the monolayer limit because of their great magnetic anisotropy. Accordingly, 2DMMs possess a vast reservoir of properties that are greatly different from their bulk counterparts, including but not limited to the ones shown in Figure 1(a), which provide an ideal platform to explore the fundamental physics for the future application of 2DMM devices. For instance, (1) owing to the ultrathin thickness, 2DMMs show strong quantum confinement and mechanical flexibility; (2) 2DMMs possess good sensitivity and responsibility to defect engineering and external stimuli because the most atoms are exposed at the surface; (3) 2DMMs can be artificially and flexibly integrated into various heterostructures on arbitrary substrates; (4) 2DMMs also show many thickness-dependent, highly anisotropic, and multiphysical field tuning properties. Although the investigation of 2DMMs is still at the primary stage, there have already been reported abundant ferromagnetic or antiferromagnetic ones, exhibiting quite different magnetic properties compared with their bulk counterparts. For instance, VSe_2_ is paramagnetic in the bulk form but shows a ferromagnetic order in the monolayer limit. Interestingly, the ferromagnetic order of VSe_2_ can persist up to room temperature, and this phenomenon also exists in MnSe_2_ and Fe_5_GeTe_2_, etc. Figure 1(b), summarizes the values of transition temperatures and critical fields for various representative 2DMMs. These values are dependent not only on the material components but also on the layer numbers or crystal thicknesses, which is beneficial to meet different demands in the device design and fabrication. Thus, 2DMMs are promising building blocks for next-generation information devices, such as nanoscale spintronics and quantum technologies.

In this review, we aim to review the current progresses and thoughts on 2DMMs, including their magnetic structure, physical properties, and device applications. We highlight the experimental descriptions on optical and transport properties as well as the theoretical studies. Furthermore, we outline the spintronic applications of 2DMMs by discussing several typical device configurations, such as spin valve, spin-orbit torque, spin field-effect transistors, and spin-filter magnetic tunnel junctions. At last, an outlook of the current challenges and future developments in this field is given, which may be a useful guide for theorists and experimentalists who are exploring the physical properties and spintronic devices of low-dimensional magnets.

## 2. Microscopy Characterizations

Microscopy techniques are usually used to measure and map either morphology or structure of materials, which utilize ions, electrons, photons, or physical cantilever probes to collect real-space information of the material structure at various length scales. For magnets, microscopy has been used to observe the spin arrangement, magnetic domain, and phase changes under external physical fields. Here, we discuss the studies of 2D magnetic materials by using several representative microscopy characterization techniques involving single-spin microscopy (SSM), photoemission electron microscopy (PEEM), and Lorentz transmission electron microscopy (TEM).

### 2.1. Single-Spin Microscopy

In recent years, optical detection of magnetic resonance (ODMR) [15] based on the nitrogen-vacancy (NV) center of the diamond has been developed rapidly. By combining with modern atomic force microscope (AFM) technology, the high spatial resolution at the nanoscale and ultrahigh detection sensitivity of single atomic spin, later known as the SSM [16], can be achieved. As shown in Figure 1(c), SSM is an electronics spin resonance (ESR) technology based on the optical detection, which utilizes confocal microscopy to detect the spin-dependent fluorescence intensity of the NV color center. Diamond nanocrystal containing the NV color center is embedded in the tip of an AFM probe. When the AFM tip approaches the sample surface, the local magnetic field of the sample leads to the Zeeman splitting of the energy level of the NV color center. As the excitation microwave frequency is consistent with the ESR frequency of the NV color center, the fluorescence intensity of the NV color center will decrease significantly. By detecting the fluorescence intensity of the NV color center and simultaneously recording the position of the AFM tip, the magnetic distribution of the sample can be obtained at microscale.

As a successful example, SSM has been used to investigate the magnetism of CrI_3_ with varying thicknesses [16]. As shown in Figure 1(d), the mono-, penta-, and nonalayer flakes all exhibit near-uniform magnetization of a magnitude comparable to the net magnetization of the monolayer (*σ*_*z*_^mono^ ~16 *μ*_B_/nm^2^). In contrast, magnetization almost disappears in the even number layers due to the antiferromagnetic interlayer exchange coupling. Moreover, from the SSM measurement results, it is also noticed that the change of morphology or crystal structure is always accompanied with a magnetic phase transition, indicating an apparent structural transition-induced modification of the ferromagnetic/antiferromagnetic interlayer coupling. Therefore, the successful application of SSM technique to 2D CrI_3_ provides a valuable demonstration to directly detect the magnetization behavior of 2DMM quantitatively, which is also potentially applicable to more complex 2DMM systems with various external fields in the future investigations.

### 2.2. Photoemission Electron Microscopy

PEEM is a powerful surface imaging technique with ultrahigh resolution of surface topography, chemical composition, and magnetic information by detecting the electrons emitted from the material surface [17]. When applied to 2D Fe_3_GeTe_2_ flakes, PEEM enables the observation of their varying ferromagnetic domain phases. The Fe_3_GeTe_2_ flakes exhibit a domain phase with stripe-like shapes below 230 K, which can be determined as alternating spin-down and spin-up magnetic domains [18]. This out-of-plane stripe-like domain phase can change to an in-plane multidomain phase in the temperature range of 230 to 370 K, which originates from a paramagnetic state rather than a ferromagnetic state. Specifically, the formation of the stripe domains in Fe_3_GeTe_2_ is caused by the dipolar interaction over the exchange interaction and magnetic anisotropy. Moreover, the magnetic dipolar interactions can be well controlled by reducing the size and adjusting the shape of Fe_3_GeTe_2_ flakes, because the magnetic moments at the edge can produce a stray magnetic field to improve the magnetostatic energy of the system. By aligning the magnetization vector along the flake plane and parallel to the edge of the microstructure, the surface magnetic charge can be minimized, which is favorable to compete with the increasing Heisenberg interaction. As a result, a new magnetic domain phase is constructed. The sharp edge of the microstructures will promote the reorientation of in-plane magnetization vector to form either a multidomain state (in a rectangular shape) or a magnetic vortex state (in a diamond shape), as presented in Figure 1(e). Considering that the surface atoms may play an important role in the magnetic behavior of 2DMMs, PEEM is a powerful tool to visually demonstrate the mapping and evolution of their magnetic domains at nanoscale.

### 2.3. Lorentz Transmission Electron Microscopy

Lorentz TEM is widely employed to analyze the microstructures of magnetization reversals and magnetic domains in magnetic thin films. Here, we provide manifestations of Lorentz TEM in the investigation of helimagnetic materials.

Lorentz TEM is regarded as an efficient method to investigate the real-space imaging of a 2D lattice of skyrmions [19, 20]. A recent study demonstrates the transformation from magnetic stripe domains to magnetic skyrmion bubbles in 2D van der Waals Fe_3_GeTe_2_ crystals below Curie temperature, with the application of an out-of-plane magnetic field in the range of 0 ~ 92 mT, as shown in Figure 1(f) [21]. Interestingly, the hexagonal lattices of skyrmion bubbles are formed when the field is 60 mT at 93 K and those skyrmion bubbles remain stable after the field decreases to zero. On the other hand, a series of Lorentz TEM images of Cr_1/3_NbS_2_ thin flakes (a monoaxial chiral magnet) has given the first experimental demonstration of one-dimensional chiral soliton lattice [22]. In the presence of a magnetic field perpendicular to the chiral axis (i.e., *c*-axis for Cr_1/3_NbS_2_), the chiral helical magnetic (CHM) structure tends to transform to chiral magnetic soliton lattice (CSL) and finally evolve to the force ferromagnetic state. Uniquely, the spin helix period of CHM and CSL can be determined by the linearly and nonlinearly periodic variation of the contrast intensity, respectively, which is bright-dark-bright or dark-bright-dark as presented in Figure 1(g). It may be noted that Cr_1/3_NbS_2_ crystals can be mechanically exfoliated to thin flakes due to their layered crystal structure [23], of which the magnetic properties are desirable to be investigated at the 2D scale by Lorentz TEM or other techniques in the future.

## 3. Optical Probes

Optical probes are applicable for the characterization of structural and physical properties of materials through light-matter interaction, with advantages of simple operation and high sensitivity. Various optical characterization methods have been used to investigate the fundamental and functional aspects of 2DMMs [24]. In this section, we mainly include the discussion on Raman scattering, photoluminescence (PL) measurements, and magnetooptic Kerr effect (MOKE).

### 3.1. Raman

Raman spectroscopy, originated from Raman effect in 1928, is a conventional tool to identify the structural information and optical properties of materials. Because of its sensitivity to lattice vibration, Raman spectroscopy can be used to investigate the spin-phonon interactions and the lattice dynamics for magnetic materials.

**Figure 2:**
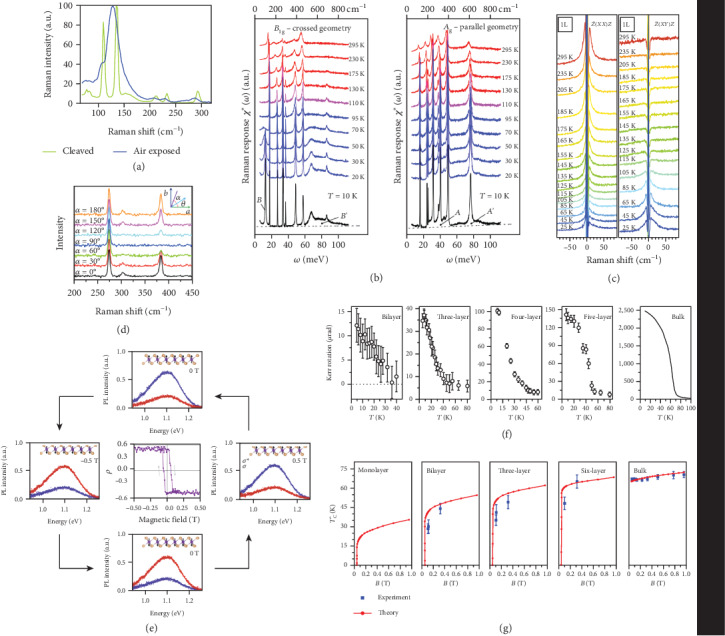
(a) Raman spectra of 30 nm Cr_2_Ge_2_Te_6_ under different conditions at 300 K. (b) Raman spectra of Ca_2_RuO_4_ with two magnetic geometries (*B*_1g_ and *A*_g_ scattering) under various temperatures. (c) Low-frequency polarized Raman spectra of monolayer NiPS_3_. (d) Polarized Raman spectra of 10 nm MnPS_3_ with *θ* = 0. (e) Photoluminescence spectra of monolayer CrI_3_ for *σ*^+^ (red) and *σ*^−^ (blue) circularly polarized photoluminescence with the application of an out-of-plane magnetic field at 15 K. The center panel is the plotting of circular polarization (*ρ*) dependence on the applied magnetic field. (f) Temperature-dependent Kerr rotation intensities of Cr_2_Ge_2_Te_6_ with varying thickness under a perpendicular field ~0.075 T. (g) Experimental (blue squares) and theoretical (red dotted lines) field-dependent *T*_C_ of Cr_2_Ge_2_Te_6_ with varying thickness. Panel (a) is reproduced with permission from ref. [26], copyright 2016 *2D Materials*. Panel (b) is reproduced with permission from ref. [27], copyright 2017 *Physical Review Letters*. Panel (c) is reproduced with permission from ref. [29], copyright 2019 *Nature Communications*. Panel (d) is reproduced with permission from ref. [31], copyright 2017 *ACS Nano*. Panel (e) is reproduced with permission from ref. [33], copyright 2017 *Nature Physics*. Panel (f, g) are reproduced with permission from ref. [13], copyright 2017 *Letter*.

For instance, Raman measurements have been conducted to observe the ambient stability and spin-phonon coupling of Cr_2_Ge_2_Te_6_ [25]. As shown in Figure 2(a), seven peaks located at around 78.6, 85.3, 110.8, 136.3, 212.9, 233.9, and 293.8 cm^−1^ can be unambiguously determined in the Raman spectra of newly cleaved Cr_2_Ge_2_Te_6_ [26]. In stark contrast, these peaks became less and broadened after exposed to ambient conditions, as a clear evidence of the oxidation and degradation of Cr_2_Ge_2_Te_6_ in air. Moreover, the temperature dependence of Raman modes can give the information on the varying interaction between the spin texture and lattice vibration in Cr_2_Ge_2_Te_6_. Below transition temperature *T*_c_ (~61 K), the splitting of two Raman peaks (78.6, 85.3 cm^−1^) in the low-energy range and the dramatic width change and position shift of three peaks (110.8, 136.3, and 212.9 cm^−1^) in the high-energy range all reveal the phase transition to a ferromagnetic state for Cr_2_Ge_2_Te_6_.

In addition, Raman spectroscopy is also powerful to investigate the spin-orbit condensate, as evidenced in a Mott insulator Ca_2_RuO_4_ with Higgs physics at the quasi-2D scale [27]. Figure 2(b) reveals the temperature-dependent Raman spectra of *A*_g_ and *B*_1g_ modes of Ca_2_RuO_4_, which split into *A* and *A*′ and *B* and *B*′ at low temperatures, respectively. In the *B*_1g_ channel, *B* is referring for single magnon excitation and *B*′ is for two-magnon scattering, while the *A*_1g_ channel enables direct detection of Higgs oscillations of soft moments.

Another case of the recent Raman studies on 2DMMs is that NiPS_3_ [28] evolves from a 2D XXZ-type antiferromagnetic system to a 2D XY system at low temperatures [29]. The magnetic signals are analyzed in the form of two-magnon and quasi-elastic scattering (QES), with the sample thickness decreasing to the monolayer limit. The Raman spectra show that when the flake is two layers or above, the peak position, intensity, and width of two magnons are nearly irrelevant with the altering of layer numbers. Nevertheless, in comparison, the monolayer sample possesses a distinct temperature dependence in Raman peaks. Moreover, the Néel temperature is slightly dependent on the layer number for all thickness expect the monolayer. All these results indicate that the antiferromagnetic ordering can exist down to bilayer flakes, but is significantly suppressed in monolayer ones. It is different from the case of FePS_3_ [30], CrI_3_, or Cr_2_Ge_2_Te_6_, which can maintain the magnetic ordering or finite Curie temperature down to the monolayer limit. In addition, from the temperature-dependent Raman spectra of monolayer NiPS_3_ shown in Figure 2(c), a low-temperature enhancement of QES due to the strong spin fluctuations can be recognized. This observation, in good agreement with theoretical predictions, confirms the XY magnetism instead of an XZZ model in monolayer NiPS_3_.

Angle-resolved polarized Raman (ARPR) spectroscopy is commonly employed to study the anisotropic properties of the materials [31, 32]. For instance, the layer-dependent Raman fingerprints and Raman peak anisotropy in antiferromagnet MnPS_3_ have been investigated by ARPR [31]. The Raman modes of MnPS_3_ gradually become weaker with decreasing thickness and finally become invisible in the monolayer case. Furthermore, the ARPR spectral measurements are performed to investigate the symmetry properties (see Figure 2(d)). The *A*_g_ Raman mode located at 383 cm^−1^ has a 100% polarity, while *B*_g_ Raman mode located at 273 cm^−1^ is independent on the crystalline orientation. Moreover, ARPR measurement is performed to study the crystal symmetry of CrCl_3_ bulk and exfoliated crystals [32]. By analyzing the Raman peak at 247 cm^−1^, it is found that there occurs a symmetry transformation from monoclinic phase (*C*2/*m* phase) at room temperature to rhombohedral phase ($R\bar{3}$ phase) at low temperature in bulk CrCl_3_. On the contrary, CrCl_3_ exfoliated crystals possess a *C*2/*m* phase at both room temperature and low temperature.

### 3.2. Photoluminescence

One step forward from Raman, PL spectroscopy is also crucial and popular in optical measurements, which probes the photoexcitation process after light absorption. The circularly polarized PL has been recently employed to the monolayer and bilayer of CrI_3_ [33]. For bilayer samples, vanished circular polarization is deduced from the two polarized PL curves (*σ*^+^ and *σ*^−^), resulting from the anomalous antiferromagnetic interlayer interaction of bilayer CrI_3_. However, for monolayer samples, the *σ*^+^ and *σ*^−^ PL spectra can be clearly distinguished below *T*_C_, with the *σ*^+^ component being stronger than *σ*^−^. As shown in Figure 2(e), an out-of-plane magnetic field is applied to the monolayer CrI_3_ to observe its PL evolution with external fields. A magnetic field of +0.5 T makes a reverse case, i.e., the *σ*^−^ (blue spectra) component being stronger than *σ*^+^ (red spectra), which originates from the magnetic field-induced magnetization flip. The PL spectra keep unchanged when the field is decreased from +0.5 T to 0 T. Further decreasing the field to −0.5 T causes the polarization flip again, leading to the stronger *σ*^+^ component than *σ*^−^. This state persists as the external field returns to zero. The PL circular polarization over a cycle evolution of the magnetic field exhibits a hysteresis loop, suggesting the typical characteristic of ferromagnetic behavior. These results demonstrate that the ligand-field transition has significant meanings in the optical investigations of 2D semiconductors with the magnetic order.

### 3.3. Magnetooptic Kerr Effect

The MOKE is a magnetooptical effect, which represents the change of the polarization of incident light when reflected (or transmitted) by a magnet [34]. MOKE is mostly used to detect and investigate the physical properties of magnetic materials, such as the magnetic domain structures, spin density of states, and magnetic phase transition dynamics. It should be noted that the intrinsic ferromagnetism of the first two 2D van der Waals magnets—i.e., CrI_3_ and Cr_2_Ge_2_Te_6_—has been firstly discovered through MOKE measurements [13, 14].

The temperature-dependent Kerr rotation of few-layer and bulk Cr_2_Ge_2_Te_6_ is shown in Figure 2(f) [13]. The emergence of ferromagnetic phase in Cr_2_Ge_2_Te_6_ can be identified through the dramatic rise of Kerr rotation as the temperature is below *T*_C_. There is a monotonic increment in *T_C_* with sample thickness increasing from bilayer (~30 K) to bulk crystals (~68 K), in good agreement with calculated values as shown in Figure 2(g). Besides, it is also found that the introduction of a small magnetic field can cause a remarkable change of *T*_C_ for the bilayer, trilayer, and six-layer samples, which is in stark contrast to the independent *T*_C_ with magnetic fields for bulk crystals.

Similar investigations have also been performed on atomically thin CrI_3_ [14]. It is found that the *T*_C_ for trilayer and multilayer CrI_3_ is ~61 K. The relatively small downturn of *T*_C_ from bulk to monolayer indicates that the ferromagnetic order in CrI_3_ is not dominated by interlayer interactions. Besides, the bilayer samples exhibit a significantly different magnetic property from the monolayer and trilayer samples, which is a consequence of the out-of-plane magnetization.

## 4. Electronic Properties

The electronic properties of 2DMMs are strongly affected by their magnetic order. In this section, we summarize the recent progresses of the electronic transport studies of 2DMMs in the aspects of the longitudinal resistance and transversal resistance (namely, the Hall resistance). In particular, the experimental investigations on the quantized anomalous Hall effect, as very recently observed in a series of 2D MnBi_x_Te_y_, are also involved.

### 4.1. Longitudinal Resistance

**Figure 3:**
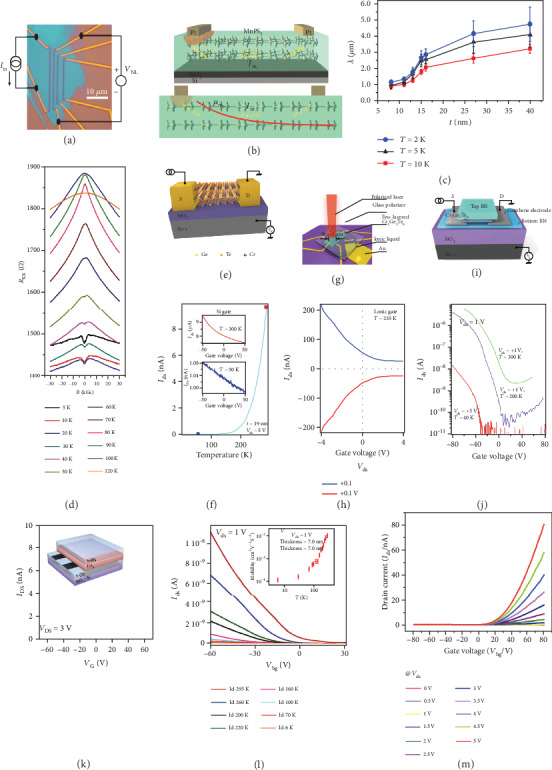
(a) Scheme of nonlocal measurement applied in a MnPS_3_ device. (b) Scheme of long-distance magnon transport of MnPS_3_. (c) Magnon relaxation distance vs. crystal thickness in quasi-2D MnPS_3_ at 2, 5, and 10 K. (d) Magnetoresistance behavior of 20 nm (Ga,Mn)As in the presence of an out-of-plane magnetic field under various temperatures. (e–j) Device configuration (e, g, and i) and transport results (f, h, and j) of Cr_2_Ge_2_Te_6_ field-effect transistor devices by using SiO_2_, ionic liquid, and boron nitride as dielectric layer, respectively. (k–m) Transfer characteristics of the field-effect transistors based on ~7 nm CrI_3_ at room temperature (k), ~7 nm CrSiTe_3_ in the temperature of 295 K down to 6 K (l), and~6.5 nm NiPS_3_ at 298 K (m). Panels (a–c) are reproduced with permission from ref. [36], copyright 2019 *Physical Review X*. Panel (d) is reproduced with permission from ref. [37], copyright 2019 *Advanced Electronic Materials*. Panels (e–j) are reproduced with permission from ref. [38], copyright 2018 *Nature Nanotechnology*. Panel (k) is reproduced with permission from ref. [39], copyright 2017 *Nature Communications*. Panel (l) is reproduced with permission from ref. [40], copyright 2016 *Journal of Materials Chemistry C*. Panel (m) is reproduced with permission from ref. [41], copyright 2018 *Scientific Reports*.

One of the exciting transport phenomena of 2DMMs is their magnonics or magnon-based spintronics, which, in the combination of magnetism and wave, mainly refer to the dynamic behavior of spin waves in nanoscale materials [35, 36]. Such magnon-mediated transport investigation has been conducted in 2D antiferromagnetic MnPS_3_ [36], as the device structure is shown in Figure 3(a). The long-distance magnon transport in MnPS_3_ is schematically illustrated in Figure 3(b), where the left Pt electrode acts as a magnon injector, while the right Pt acts as a magnon detector. A systematic measurement of the thickness-dependent magnon transport and relaxation properties of MnPS_3_ is then performed at temperatures of 2, 5, and 10 K, as shown in Figure 3(c). The reduction of the crystal thickness leads to the decrease of the magnon diffusion length, suggesting that the surface impurities in thinner flakes may induce a stronger scattering effect. As the temperature goes down, the magnon diffusion length—with the amplitude of several micrometers—becomes longer, as an indication of longer-lived magnons at low temperatures.

Magnetoresistance (MR), referring to the resistance change in the presence of a magnetic field, is a significant physical quantity to reveal the spin-order effect on the electron scattering. Figure 3(d) shows the temperature-dependent magnetoresistance behavior of 2D ferromagnets (Ga,Mn)As, which are grown by low-temperature molecular beam epitaxy (MBE) [37]. At low temperature, two distinct peaks due to the anisotropic MR is observed, where MR is positive at low fields and negative at high fields. The forced parallel alignment of spins at sufficiently high fields gives rise to the reduced electron scattering with spin disorder and thus negative MR. The “two-peak” behavior gradually fades away with increased temperature and eventually vanishes above *T*_C_.

Besides the magnetic fields, the resistance of 2DMMs can also be efficiently modulated by external electric fields in the form of applied gate voltage when fabricated into field-effect transistor (FET) devices [38]. In general, there are three representative device configurations for 2D material-based FETs, in which SiO_2_, ionic liquid, and h-BN are used as the dielectric layers, respectively. These three FET configurations based on few-layered ferromagnetic semiconductor Cr_2_Ge_2_Te_6_ are schematically illustrated in Figures 3(e), 3(g), and 3(i), respectively. Figure 3(f) shows the gate-tunable resistance behavior of a 19 nm Cr_2_Ge_2_Te_6_ flake for the first configuration. The current *I*_ds_ decreases logarithmically as the temperature decreases, indicating a semiconductor-insulator transition at around 150 K. As shown in the inset of Figure 3(f), the resistance of the flake can be weakly tuned by the back-gate voltage at low temperatures, of which the effect is more evident at high temperatures. As for the second FET configuration with the formation of an electric double layer (EDL) in the ionic liquid, the gate tunability is greatly enhanced, as shown in Figure 3(h). At *T* = 235 K, the ratio of the gate-tuned resistance is up to 10 when the gate voltage sweeps from −4 to 4 V. The optimal gate-tunable electronic behavior is accomplished in the BN-based FET configuration, as shown in Figure 3(j). This is evidenced by the much higher on/off ratio and the observed bipolar transport behavior.

In addition, there are some investigations of other 2DMM FET devices, as shown in Figures 3(k)–3(m), all of which are based on back-gate configurations using SiO_2_ as a dielectric layer. For instance, the gate-dependent current of few-layer CrI_3_ is presented in Figure 3(k), from which the *n*-type semiconductor nature of CrI_3_ is revealed by the conduction channel opening at a positive gate voltage [39]. Different from CrI_3_, the *I* − *V* characteristics of CrSiTe_3_ measured at various temperatures indicate a *p*-type semiconductor behavior, as shown in Figure 3(l) [40]. Also, the mobility of CrSiTe_3_ increases with elevating temperature, similar to that of monolayer MoS_2_. Another case is NiPS_3_ (see Figure 3(m)), showing nonlinear *I* − *V* curves because of the formation of a Schottky barrier with contacting Au electrodes [41]. Since the current increases with the positive gate voltage, NiPS_3_ is also proven to be a *n*-type semiconductor like CrI_3_.

It is well known that, because of Thomas–Fermi screening, the tuning effect of electric fields can only apply to a maximal channel depth of several nanometers. In this sense, 2D materials with ultrathin thickness are appealing for their full gate tunability. Moreover, it is highly desirable that the gate voltage can induce great effect on the charge carriers and spin arrangements simultaneously, with the creation and modulation of more electronic and magnetic phases. Therefore, 2DMMs stand a chance of manifesting themselves in the future FET or spin-FET devices of high-efficient performance and new working modes, the realization of which surely requires much more research efforts.

### 4.2. Anomalous Hall Effect

**Figure 4:**
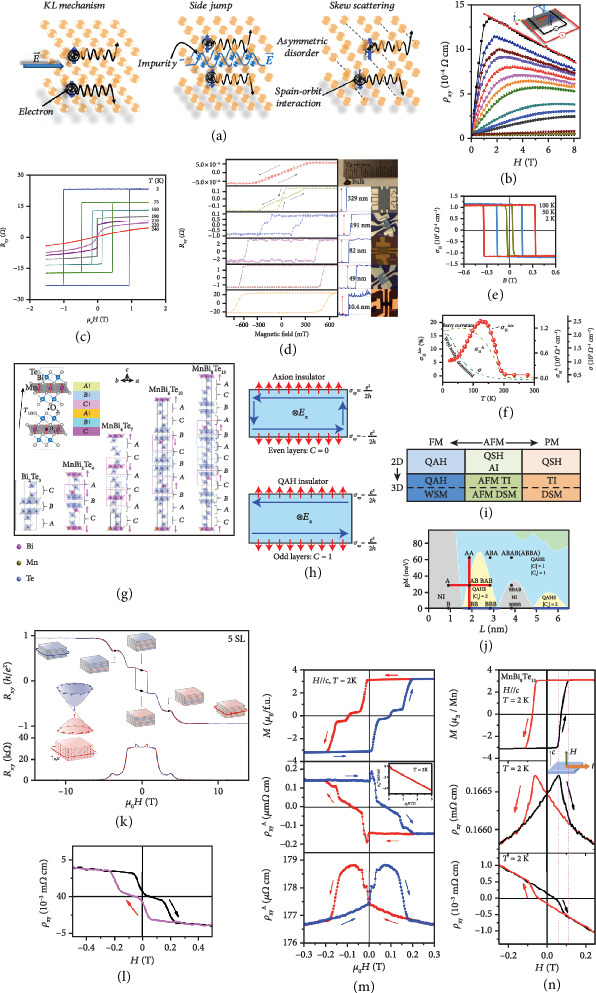
(a) Scheme of three basic mechanisms of anomalous Hall effect. (b) Hall resistivity of Cr_0.68_Se at 2 K. (c, d) Hall resistivity of Fe_3_GeTe_2_ of 12 nm thick under various temperatures (c) and of varying thicknesses at 2 K (d) with *H*//*c*. (e, f) Hall resistivity of Co_3_Sn_2_S_2_ as a function of out-of-plane magnetic field at 100, 50, and 2 K (e) and as a function of temperature at zero magnetic field (f). (g) Scheme of the crystal lattice and magnetic structure of MnBi_2n_Te_3n+1_ family (*n* = 0, 1, 2, 3, ⋯). (h) Illustration of intrinsic axion insulator in even layers and quantum anomalous Hall (QAH) insulator in odd layers. (i) Topological phases and magnetic phases of 2D and 3D MnBi_2_Te_4_. (j) Topological phase diagram in the 2D limit of a 3D topological insulator as a function of the crystal thickness *L* and magnetization gM, including the normal insulator (grey area), quantum spin Hall effect (QSHE) with preserved time-reversal symmetry (blue line), QSHE with broken time-reversal symmetry (yellow area), and quantum anomalous Hall effect (QAHE) (cyan area). (k) Magnetic transition in five-layer MnBi_2_Te_4_ under *H*//*c* and *H*//*ab* at 1.6 K. (l) Hall resistivity of bulk MnBi_4_Te_7_ at 2 K with *H*//*c* and *I*//*ab*. (m, n) Magnetization, Hall resistivity, and planar magnetoresistivity of few-layer MnBi_6_Te_10_ with *H*//*c* of ±0.3 T (m) and MnBi_8_Te_13_ with *H*//*c* of ±0.2 T (n) at 2 K. Panels (a, b) are reproduced with permission from ref. [43], copyright 2017 *Applied Physics Letters*. Panel (c) is reproduced with permission from ref. [46], copyright 2018 *Nature Materials*. Panel (d) is reproduced with permission from ref. [47], copyright 2018 *Nature Communications*. Panels (e, f) are reproduced with permission from ref. [48], copyright 2018 *Nature Physics*. Panels (g, n) are reproduced with permission from ref. [59], copyright 2019 *Arxiv*. Panels (h, i) are reproduced with permission from ref. [54], copyright 2019 *Science Advances*. Panel (j) is reproduced with permission from ref. [57], copyright 2019 *Physical Review Letters*. Panel (k) is reproduced with permission from ref. [55], copyright 2019 *Arxiv*. Panel (l) is reproduced with permission from ref. [56], copyright 2020 *Nature Communications*. Panel (m) is reproduced with permission from ref. [58], copyright 2019 *Arxiv*.

Anomalous Hall effect (AHE) is an important physical phenomenon occurring in the materials with broken time-reversal symmetry. It is fundamentally different from the ordinary Hall effect (that only involves the orbital deflection of the carrier transport caused by the external magnetic field-induced Lorenz force) [42]. Basically, there are three basic mechanisms to induce AHE, as shown in Figure 4(a) [43]. The AHE can either be intrinsic or extrinsic. The intrinsic AHE is determined by a geometric phase factor (Berry phase) as the wave function of an electron going throughout the crystal. The Karplus-Luttinger (KL) mechanism then offers a clear interpretation of the intrinsic AHE, in which the external electric field induces an anomalous group velocity (namely, Berry phase curvature) of electrons to produce AHE, while the other two mechanisms give rise to the extrinsic AHE that occurs in the presence of impurity or disorder due to the spin-order-dependent electron scattering. In the side-jump mechanism, the anomalous velocity originates from the potential field of impurity or disorder, but the skew-scattering mechanism ascribes the velocity to the asymmetric scattering of impurity or disorder. The first two mechanisms follow the same scaling relation *ρ*_*xy*_^*A*^ ∝ *ρ*_*xx*_^2^, where *ρ*_*xx*_ and *ρ*_*xy*_^*A*^ are longitudinal resistance and abnormal transversal resistance, respectively [44]. However, the skew scattering follows a different relation *ρ*_*xy*_^*A*^ ∝ *ρ*_*xx*_.

It has been demonstrated that the AHE in 2D Cr_0.68_Se, with a Néel temperature around 42 K, is dominated by the skew-scattering mechanism rather than the side-jump or KL mechanism [43]. The temperature-dependent transversal resistance *ρ*_*xy*_ of Cr_0.68_Se is shown in Figure 4(b). As can be seen, the measured *ρ*_*xy*_ exhibits a huge kink of decreasing at low fields and then increasing at high fields, confirming the existence of AHE. The intrinsic AHE in 2D ferromagnets has also been experimentally investigated in Fe_3_GeTe_2_ [45, 46]. Figure 4(c) shows the temperature-dependent AHE of a 12 nm thick flake. Under the applied magnetic field along the *c*-axis, this hysteresis is almost in perfect rectangular shape at low temperatures. The coercive field is 1 T at around 2 K, then decreases with the rising temperature, and eventually vanishes above *T*_C_ (~210 K), which reflects the characteristic of ferromagnetic Fe_3_GeTe_2_ with strong out-of-plane anisotropy. Besides, there are experimental results showing that the AHE of Fe_3_GeTe_2_ is strongly dependent on its thickness [47]. A hard-magnetic phase with large coercivity and almost rectangular-shaped hysteresis loop occurs in the samples less than 200 nm thick, as shown in Figure 4(d). Specifically, as the thickness decreases, the 329 and 191 nm samples exhibit similar magnetic loops with the coexistence of “two phases”—soft phase (bulk) and hard phase (layers) containing two different coercivity values. Nevertheless, the “two-phase” phenomenon vanishes as the thickness further decreases. The 82, 49, and 10.4 nm samples exhibit a nearly square shape of hysteresis loops, the feature of one single hard magnetic phase, and possess larger coercivities than the 329 and 191 nm samples. Also, the 191, 82, 49, and 10.4 nm samples exhibit the *M*_R_/*M*_S_ ratios (the hard magnetic phase divided by large magnetic remanence to saturated magnetization) of ~1 at 2 K, indicating that all the magnetic moments are arranged along the *c*-axis at the remanence point. This work also confirms that the interlayer coupling length in Fe_3_GeTe_2_ is around five layers.

AHE also occurs in magnetic Weyl semimetals, such as Co_3_Sn_2_S_2_ with kagome lattices, where a strong AHE is proven to be induced by its topology of Weyl bands [48]. As shown in Figure 4(e), rectangular-shaped hysteresis loops are discovered in Co_3_Sn_2_S_2_, similar to those of Fe_3_GeTe_2_ discussed above. The coercive field significantly increases with the decreased temperature, suggesting a giant remanent Hall resistivity in Co_3_Sn_2_S_2_ at zero field. Besides, the plot of Hall angle *σ*_*H*_^*A*^/*σ* (the ratio between the anomalous Hall conductivity to charge conductivity at zero magnetic field) versus the temperature is given in Figure 4(f). A large value as much as 20% of *σ*_*H*_^*A*^/*σ* is observed around 120 K, mainly due to the topological protection of band. All these observations suggest that magnetic Weyl semimetals offer a good system to combine topological phases and magnetic interactions together, which may lead to new physics and modern technologies in the field of electronics and spintronics. Furthermore, Co_3_Sn_2_S_2_ is also predicted to be an excellent system for the study of quantum anomalous Hall state in the 2D limit that may need further research effort.

Quantum anomalous Hall effect (QAHE) is an exotic transport phenomenon that only emerges in magnetic topological insulators [49]. In the quantum anomalous Hall insulator, the spontaneous magnetic moments integrated with spin-orbit coupling give rise to a topologically nontrivial band structure exhibiting QAHE in the absence of a magnetic field [50]. A Cr-doped (Bi,Sb)_2_Te_3_ thin film is the first magnetic topological insulator that experimentally demonstrates QAHE [51]. More recently, several transition metal ternary tetradymite materials in the 2D limit have been proven to intrinsically possess the characteristics to reveal QAHE, as the crystal structures are schematically shown in Figure 4(g). For example, 2D MnBi_2_Te_4_, with in-plane ferromagnetic order and out-of-plane antiferromagnetic order, presents an evolution of the magnetic properties and topological phases with varying layer numbers [52, 53]. As shown in Figure 4(h), for even layer numbers, MnBi_2_Te_4_ become an axion insulator due to the vanishing net magnetization, while for odd layer numbers, they are ideal candidates of a Chern insulator to realize QAHE [54]. This is because the magnetization between the top layer and the bottom layer is in the opposite (same) direction for even (odd) layers, which results in *C* = 0 (*C* = 1). Rich topological phases of MnBi_2_Te_4_, including both 2D and 3D structures, are summarized in Figure 4(i). Among them, the states of ferromagnetism, paramagnetism, and antiferromagnetism have already been realized by tuning extremal fields and temperatures. A five-septuple layer of MnBi_2_Te_4_ enables the observation of zero-field QAHE at the temperature of 1.4 K as shown in Figure 4(k) [55]. While an external magnetic field assists to align all the layers ferromagnetically, as a result, the temperature for the observation of QAHE is increased to 6.5 K [53].

It is further found that more members belonging to the MnBi_x_Te_y_ family are potential materials to reveal QAHE or quantum spin Hall effect [56]. For instance, MnBi_4_Te_7_ with a transition temperature around 13 K exhibits a ferromagnetic alignment at 0.2 T, one order smaller than that of MnBi_2_Te_4_, implying that their ferromagnetic states can more easily achieve for the observation of AHE. The AHE results of MnBi_4_Te_7_ are shown in Figure 4(l). Similar members such as MnBi_6_Te_10_ and MnBi_8_Te_13_ are also reported with a fascinating Hall effect, as shown in Figures 4(m) and 4(n), respectively.

Therefore, the discovery of more MnBi_x_Te_y_ members indicates that they can work complementarily and synthetically with each other to reveal various topological states. By comparing Figures 4(k) and 4(l), the spin-flip transition in MnBi_2_Te_4_ starts at ~3.5 T, while the first flip in MnBi_4_Te_7_ takes place at ~0.15 T for the same temperature at ~2 K [56]. Moreover, the saturation field of MnBi_4_Te_7_ at ~0.22 T demonstrates the weaker interlayer antiferromagnetic exchange interaction than that of MnBi_2_Te_4_. As the temperature increases up to 10 K, the hysteresis of MnBi_4_Te_7_ vanishes with the little change of the coercivity, which suggests a dramatic spin-flipping in the temperature range of 10 K and *T*_N_. On the other hand, MnBi_6_Te_10_ crystals can maintain the polarized ferromagnetic phase in the absence of a magnetic field, and this phase reduces when the field is 0.01 T at 2 K [57]. This value of the required magnetic field is much smaller than that of other two family members—MnBi_2_Te_4_ and MnBi_4_Te_7_. Furthermore, the hysteresis loop shows two plateaus at ±0.1 T, indicating that MnBi_6_Te_10_ gets into the antiferromagnetic state between the two ferromagnetic states, while in MnBi_8_Te_13_, the topological axion state shows the strong coupling of charge carriers and magnetism with similar electron carrier density to that of MnBi_2_Te_4_ [58].

Theoretical researchers also make a big effort to provide abundant information on how to design these topological materials for QAHE investigation. Figure 4(j) is a topological phase diagram for the MnBi_x_Te_y_ family, as a function of the magnetization gM and the crystal thickness *L* [59]. In consideration of this, more experimental work needs to be devoted to realize these various topological states by exploring the superlattice structures of the MnBi_x_Te_y_ family in terms of crystal thickness, interlayer distance, and magnetization strength.

## 5. Spintronic Applications

**Figure 5:**
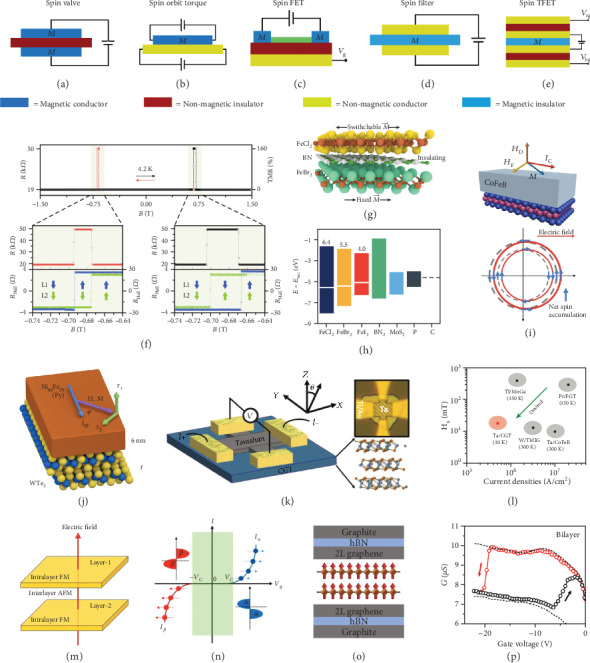
(a–e) Schematic illustrations of representative spintronic devices: spin valve (a), spin-orbit torque (b), spin field-effect transistor (c), spin-filter magnetic tunnel junction (d), and spin tunnel field-effect transistor (e). (f) Spin valve effect in a Fe_3_GeTe_2_ (~7 nm)/hBN/Fe_3_GeTe_2_ (~20 nm) with corresponding tunneling resistance and Hall resistance at 4.2 K with an applied field perpendicular to the layers. (g) Scheme of a magnetic tunnel junction based on 2D FeCl_2_ (top), 2D BN (middle), and 2D FeBr_2_ (bottom). (h) Spin gaps and Fermi levels of FeCl_2_, FeBr_2_, FeI_2_, BN, MoS_2_, black phosphorene (P), and graphene (C). (i) Measurement setup of spin-orbit torque (upper) and illustration of induced spin accumulation (lower) based on MoS_2_ or WSe_2_ (monolayer)/CoFeB (3 nm) at 300 K. (j) Scheme of the sample geometry of spin-orbit torque based on WTe_2_ (5.5 nm)/permalloy (6 nm) at room temperature. (k) Measurement setup and device configuration based on Cr_2_Ge_2_Te_6_ (50 nm)/tantalum (5 nm) spin-orbit torque. (l) The previous reported values of current density and in-plane field for spin-orbit torque switching. (m) Schematic diagram of the A-type antiferromagnetic in bilayer system with an electric field from layer 2 to layer 1. (n) Schematic spin-polarized current as a function of the gate voltage. (o) Schematic diagram of a spin tunnel field-effect transistor based on graphite (few-layer)/h-BN (~20 nm)/graphene (bilayer)/CrI_3_ (bilayer)/graphene (bilayer)/h-BN (~20 nm)/graphite (few-layer). (p) Gate-tunable tunneling conductance of a bilayer CrI_3_-based TFET at 2 K and 0.76 T. Panel (f) is reproduced with permission from ref. [60], copyright 2018 *Nano Letters*. Panels (g, h) are reproduced with permission from ref. [61], copyright 2017 *Nano Letters*. Panel (i) is reproduced with permission from ref. [64], copyright 2016 *Nano Letters*. Panel (j) is reproduced with permission from ref. [65], copyright 2016 *Nature Physics*. Panels (k, l) are reproduced with permission from ref. [66], copyright 2020 *Advanced Materials*. Panels (m, n) are reproduced with permission from ref. [67], copyright 2018 *PNAS*. Panels (o, p) are reproduced with permission from ref. [68], copyright 2019 *Nature Electronics*.

The extraordinary properties possessed by 2DMMs manifest themselves in the promising potential for future applications. One attractive aspect of 2DMMs is that electrical routes can efficiently control their magnetization. Therefore, through the utilization of external electric fields, researchers have devoted their effort to tune not only the charge transport but also the spin transport, known as spintronics. Basically, what makes spintronics different from electronics is that the electron spin is explored as a further degree of freedom, leading to high efficiency in data storage, transfer, and computation. Based on 2DMMs, many spintronic devices have been successfully realized in experiments, including spin valves, spin-orbit torques, spin field-effect transistors, and spin filters, whereas a 2D spin tunnel field-effect transistor is also promising as predicted by theory. Therefore, this section will mainly focus on these spintronic applications, as schematically illustrated in Figures 5(a)–5(e).

### 5.1. Spin Valve

A spin valve is a device composed of two or more layers of conducting magnetic materials separated by nonmagnetic insulators, whose low- or high-electrical resistance states can be tuned by the magnetization alignment of each layer in parallel or antiparallel, respectively.  Figure 5(a) illustrates the simplest case, in which a nonmagnetic insulator is sandwiched by two conducting magnetic materials.

Such a simple spin-valve device has been experimentally realized using conducting ferromagnet Fe_3_GeTe_2_ [60]. In the device of a Fe_3_GeTe_2_/hBN/Fe_3_GeTe_2_ heterostructure, the tunneling resistance shows a typical behavior of spin valve with a maximum (minimum) value when the magnetization of two electrodes is antiparallel (parallel) to each other, as shown in Figure 5(f). The tunneling magnetoresistance can reach up to ∼160% at *T* = 4.2 K, corresponding to a 66% spin polarization. As the temperature increases, the spin polarization evolution (extracted from the tunneling magnetoresistance) is in good accordance with the temperature-dependent magnetization (extracted from the anomalous Hall conductivity analysis).

A 2D spin valve based on iron dihalide materials, namely, FeCl_2_, FeBr_2_, and FeI_2_, is theoretically predicted, for which a typical device is schematically shown in Figure 5(g) [61]. This magnetic material family is extremely interesting due to the characteristic of half metals with significant spin gaps. Half metals refer to the materials possessing insulating states of electrons with opposite spin polarization and metallic states of singular spin-polarized electrons. FeCl_2_ and FeBr_2_ own direct spin gaps, while FeI_2_ has indirect one. The spin gap values of FeCl_2_, FeBr_2_, and FeI_2_ are shown in Figure 5(h), with the comparison to some conventional nonmagnetic 2D materials. In particular, FeCl_2_ has the largest spin gap among all the reported 2D materials, and its predicted transition temperature is above 100 K. These 2D half-metallic materials with large spin gap are ideal candidates for spin injection/detection contacts in diverse spintronic applications such as spin FET discussed later.

### 5.2. Spin-Orbit Torque

Spin-orbit torque (SOT) is a new method for the manipulation of the magnetization in ferromagnetic materials by injecting an in-plane electric current via a large spin-orbit coupling [62]. In a SOT device (see Figure 5(b)), the current flowing through a bilayer system composed of a heavy metal and a ferromagnet can undergo a serious movement in the generated magnetization. The switching of magnetization in conventional SOT bilayer systems depends on both the intrinsic spin Hall effect and the quality of the interface [63]. The present 2D SOT devices involve two cases, in which either the heavy metal or the ferromagnet layer in conventional SOT bilayer systems is made from 2D materials.

A SOT device consisting of transition metal dichalcogenide monolayers (MX_2_: M=Mo, S; X=S, Se) and interfaced ferromagnetic CoFeB is demonstrated in Figure 5(i), which schematically illustrates the spin accumulation at the interface of MX_2_/CoFeB due to the Rashba-Edelstein effect under an external field [64]. The spin-splitting Fermi surface in an equilibrium state and under an applied electric field is denoted by grey dashed circles and solid red circles, respectively. In such SOT device, the robust spin conductivity induced by the Rashba-Edelstein effect is not sensitive to the varying temperature, revealing that the charge-spin conversion of this 2D heterostructure is very efficient for the magnetization reversal.

In order to hold the most current-efficient type of magnetic reversal (antidamping switching), the current-induced SOT can only work for magnetic devices with in-plane anisotropy but make no contribution to the ones with out-of-plane anisotropy [65]. However, an experiment about the out-of-plane antidamping torque by employing WTe_2_/permalloy bilayers has been demonstrated to overcome this limitation, as shown in Figure 5(j). This should be ascribed to the presence of only one mirror plane and the absence of twofold rotational invariance in the crystal surface of WTe_2_. Hence, the out-of-plane antidamping torque generated by the applied current is along a low-symmetry axis instead of a high-symmetry axis. The merit of perpendicular magnetic anisotropy devices compared to in-plane-magnetized devices is that they can be densely compacted while retaining the thermal stability, offering a good strategy for future engineering of SOT devices.

More recently, a device with 2DMMs acting as the ferromagnetic layer in the bilayer SOT system has also been successfully fabricated, as shown in Figure 5(k) [66]. In this Cr_2_Ge_2_Te_6_/Ta heterostructure device, the combined in-plane magnetic field and charge current flowing through Ta are applied to manipulate the magnetization in Cr_2_Ge_2_Te_6_. Figure 5(l) gives a simple summary of previously reported SOT devices in comparison to Cr_2_Ge_2_Te_6_/tantalum device. In particular, the switching current densities of Cr_2_Ge_2_Te_6_/Ta SOT devices are much lower than those of other 3D ferromagnetic material-based devices such as CoFeB, which clearly demonstrates the benefit of 2DMMs for the realization of low-consuming and high-compacted SOT devices. Without doubt, it is more promising if both the two functional layers in a SOT device can be constructed by 2D materials. We envision that various 2D elements of such pure 2D SOT systems can influence, promote, and complement each other and finally form a joint effect to induce the dramatic changes in the performance parameters of SOT devices to eventually achieve a qualitative leap.

### 5.3. Spin Field-Effect Transistors

The applications of transistors based on spin rather than charge—which is called spin transistors—could reveal high performance in nonvolatile memory applications. However, the realization is still a big challenge. The schematic diagram of the spin FET is simply presented in Figure 5(c), in which the drain/source electrodes are made from ferromagnetic materials for the spin polarizer and analyzer of electrons along the current-flow direction. The spin FET constructed by using 2D magnet 2H-VSe_2_ has been proposed recently, for which a vertical electric field is applied with the aim of switching A-type antiferromagnet to half metallic [67]. A-type antiferromagnet shows an interlayer antiferromagnetic order and intralayer ferromagnetic order, as shown in Figure 5(m). In the absence of an electric field, the energy of spin-*α* electrons is lower than that of spin-*β* electrons in layer 1, and the case is reverse in layer 2, while the application of an electric field perpendicular through layer 2 to layer 1 would increase (decrease) the energy of the electrons for both spin-*α* and spin-*β* in layer 1 (2), as shown in Figure 5(n). Furthermore, the doping effect induced by the electric field can enhance the conductivity of 2H-VSe_2_. Although this is only a spin FET proposal, it is more than possible for the experimental realization of 2D half metals and spin FETs with the exponentially growing of 2DMMs.

One step forward from spin FETs, the spin tunneling field-effect transistor (spin TFET) has been experimentally demonstrated, of which a simple scheme is shown in Figure 5(e). Basically, spin TFET is a good combination of spin FET and spin filter. This device is based on a dual-gated graphene/CrI_3_/graphene tunnel junction, as demonstrated in Figure 5(o) [68]. CrI_3_ works as a gate-tunable tunneling barrier, which can evolve into interlayer ferromagnetic or antiferromagnetic coupling as controlled by external gate voltage. The operating principle for the spin TFET is based on the combination of a gate-tunable interlayer magnetic coupling for “writing” and a spin filtering function for “reading.” In particular, Figure 5(o) schematically shows a bilayer CrI_3_ spin-TFET configuration, and its gate-tunable tunneling conductance behavior at 0.76 T is shown in Figure 5(p). A wide variety of resistance states, as modulated by the layer numbers of CrI_3_, gate-voltages, temperatures, and magnetic fields, have been demonstrated in this work. We believe that 2D spin TFETs will have great potential for future logic devices and information applications.

### 5.4. Spin-Filter Magnetic Tunnel Junctions

**Figure 6:**
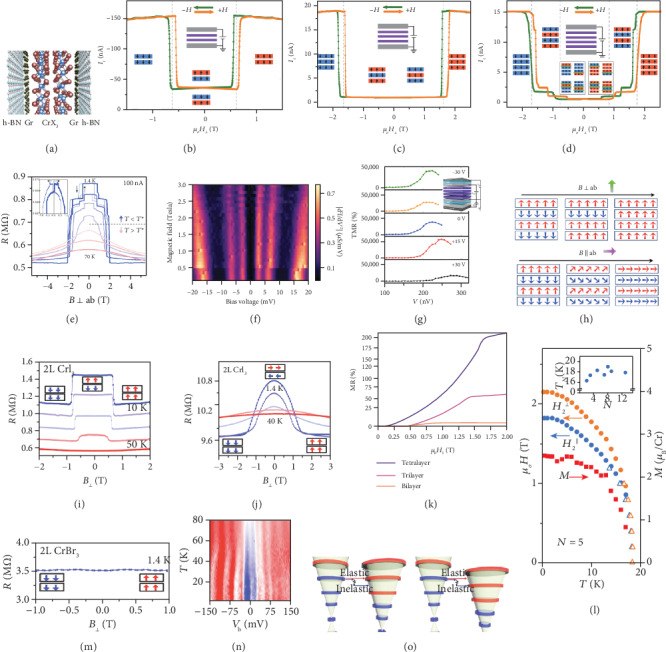
(a) Schematic illustration of magnetic tunnel junction based on 2D chromium trihalides. (b–d) Tunneling current vs. out-of-plane magnetic field of CrI_3_ bilayer (b), trilayer (c), and tetralayer (d) at 2 K. (e) Magnetoresistance of 14-layer CrI_3_ under various temperatures. (f) |*d*^2^*I*/*dV*^2^| as a function of applied magnetic field and DC bias voltage in bilayer CrI_3_ at 300 mK. (g) Tunneling magnetoresistance ratio vs. DC bias voltage of tetralayer CrI_3_ under different gate voltages at 2 K. (h) Transition mechanism of spin states in tetralayer CrI_3_ under out-of-plane and in-plane applied field, respectively. (i, j) Tunneling resistance and interlayer magnetic coupling in bilayer CrI_3_ at 10, 20, 30, 40, and 50 K (i) and bilayer CrCl_3_ at 1.4, 10, 20, 30, and 40 K (j). (k) Magnetoresistance of bilayer, trilayer, and tetralayer CrCl_3_ at 4 K. (l) Temperature-dependent spin-flip field *H*_2_^‖^ (T) (blue), *H*_2_^⊥^ (T) (orange), and layer magnetization *M* (red) for a pentalayer CrCl_3_. (m) Tunneling resistance and interlayer magnetic coupling in bilayer CrBr_3_ at 1.4 K. (n) Measured inter-Landau-level (LL) tunneling conductance as a function of temperature and DC bias in hexalayer CrBr_3_ at 17.5 T. (o) Schematic diagram of inelastic and elastic tunneling between the LLs of the two graphene layers. Panel (a) is reproduced with permission from ref. [73], copyright 2019 *Nano Letters*. Panels (b–d) are reproduced with permission from ref. [74], copyright 2018 *Science*. Panels (e, h) are reproduced with permission from ref. [75], copyright 2018 *Nano Letters*. Panel (f) is reproduced with permission from ref. [76], copyright 2018 *Science*. Panel (g) is reproduced with permission from ref. [77], copyright 2019 *Nano Letters*. Panels (i, j, m) are reproduced with permission from ref. [78], copyright 2019 *PNAS*. Panel (k) is reproduced with permission from ref. [32], copyright 2019 *Nature Physics*. Panel (l) is reproduced with permission from ref. [80], copyright 2019 *Nature Nanotechnology*. Panels (n, o) are reproduced with permission from ref. [35], copyright 2018 *Nature Electronics*.

The spin filter is a fundamental component in magnetic multilayer devices for the creation and modulation of spin-polarized currents. As shown in Figure 5(d), it consists of a magnetic insulator as a tunneling barrier, in which the spins switch between spin-up and spin-down, with efficient control on the tunneling transport in vertical junctions [39, 69, 70]. The assembly of 2D materials in various heterostructures has offered many possibilities of unique properties and fascinating functionalities. For 2DMMs, this artificial stacking technique has also well demonstrated excellent switching characteristics from electronics to spintronic devices [71, 72], such as spin-filter magnetic tunnel junctions (sf-MTJs). Among the reported 2D magnetic tunnel junctions, a material family called chromium trihalides is the most popular candidate as a tunneling barrier [73]. A typical device configuration is schematically illustrated in Figure 6(a). It is found that the tunneling magnetoresistance can be efficiently controlled or manipulated in multiple manners, i.e., the material component, the number of layers [32, 74], the environment temperature [75], the external magnetic field along different directions [75], the applied DC bias [76], and gate voltage [77].

CrI_3_ is mostly explored among this material family for spin-filter applications. An investigation of the tunneling magnetoresistance of CrI_3_ with various numbers of layers (bilayer, trilayer, and tetralayer) is conducted, as shown in Figures 6(b)–6(d) [74]. It is noted that the common feature in ferromagnets is that the out-of-plane magnetization is smaller than in-plane magnetization, which gives rise to anisotropic magnetoresistance (MR). In the case of bilayer CrI_3_, its antiferromagnetic structure is beneficial to offer a naturally formed spin filter between the two layers, which is vital for achieving large spin-filter tunneling magnetoresistance (sf-TMR). Furthermore, the interlayer coupling in trilayer CrI_3_ can be determined as antiferromagnetic, and the ground-state net magnetization is around 1/3 of the saturated magnetization when the applied field is sufficiently large to fully align all the spins of the three layers. With further increasing of the CrI_3_ thickness beyond three layers, more complicated magnetic configurations are formed. Each layer acts as a spin filter and aligns oppositely in series, which dramatically enhances the sf-TMR with the increased layer number. These sf-MTJs, originating from the intrinsic layer-by-layer magnetic ordering of CrI_3_, reveal that the layered antiferromagnets are potential for engineering multiple magnetoresistance states in an individual sf-MTJ.

On the other hand, the tunneling magnetoresistance of CrI_3_ can also be tuned by temperature, as shown in Figure 6(e) [75]. The ten-layered CrI_3_ exhibit various magnetoresistance behavior at different temperatures, with the emergence of sudden jumps in the presence of a weak magnetic field at low temperatures. These jumps are accompanied by hysteresis between different sweep directions. The |*d*^2^*I*/*dV*^2^| characteristics as a function of the applied magnetic field and bias voltage are shown in Figure 6(f) [76]. There are three peaks (3, 7, and 17 meV at zero field) on both positive and negative sides of bias voltage. The peaks originate from inelastic electronic tunneling, which would appear when the tunneling energy (eV_DC_) is larger than the collective excitation energy of the electrodes or barrier. The energy of all the three peaks linearly increases with the applied field, as a consequence of Zeeman effect and magnon renormalization effect. In addition, the magnetic states in CrI_3_ also can be induced by the gate voltages, which become a sf-MTJ configuration as discussed above [77]. The TMR ratio under various back gate voltages is shown in Figure 6(g). This phenomenon is caused by a combined effect of electric field-induced changes in the Fermi level of graphene electrodes and in the spin transitions of tunneling barrier CrI_3_. Another way to manipulate the value of TMR is through modulating the spin coupling by applying magnetic field along different directions [75]. Figure 6(h) demonstrates the possibilities of multiple combinations of different interlayer spin coupling to realize a large and varying TMR.

To compare the tunneling resistance of different material members of chromium trihalides, the MR with the application of a perpendicular magnetic field under various temperatures for CrI_3_, CrCl_3_, and CrBr_3_ are shown in Figures 6(i), 6(j), and 6(m), respectively [78]. In the case of bilayer CrI_3_, the spin-flip transforms from an antiferromagnetic to ferromagnetic state with the increasing field indicated by the dramatic reduction of resistance, as shown in Figure 6(i). The resistance drops vanish when the temperature increases up to the transition temperature. The similar behavior occurs in bilayer CrCl_3_, as shown in Figure 6(j). This indicates that the interlayer coupling in CrCl_3_ is antiferromagnetic like the case of CrI_3_ [79]. The TMR behavior of CrCl_3_ with various layer numbers is presented in Figure 6(k), with a clear demonstration of the layer number dependence of both the TMR amplitude and the transition critical field [32]. This suggests the increase of the interlayer coupling strength with the increasing thickness of CrCl_3_ up to few layers. Meanwhile, the temperature-dependent spin-flip field of a pentalayer CrCl_3_ is shown in Figure 6(l) [80]. Below the transition temperature, the values of the required magnetic field for spin flip, either the field direction along or perpendicular to the *c*-axis, increase with the decreasing temperature. Interestingly, the difference between the values of transition magnetic fields for the two cases (along or perpendicular to the *c*-axis) can give the value of the layer magnetization, as shown in the right *y*-axis in Figure 6(l). It has been theoretically proven that the magnetization value is corresponding to the sublattice magnetization of a single layer at zero field. It is of great significance that the temperature dependence of the sublattice magnetization of CrCl_3_ can be obtained through a magnetotransport measurement method. A full magnetic phase diagram of CrCl_3_, i.e., how the magnetic tunneling resistance varies with the layer number, temperature, and applied magnetic field, is experimentally provided.

Different from the description on CrI_3_ and CrCl_3_ [78], in the case of bilayer CrBr_3_, the spins of both layers naturally align in the ferromagnetic state, and thus, the low-temperature resistance remains unchanged with the varying magnetic field (the direction is perpendicular to the layers), as shown in Figure 6(m). Further exploration in CrBr_3_ is the study of magnon tunneling based on a graphene/CrBr_3_/graphene junction device [35]. Under the application of a perpendicular magnetic field, the tunneling conductance between the Landau levels (LLs) formed in graphene and through a six-layer CrBr_3_ barrier, as a function of the applied voltage bias and temperature, is shown in Figure 6(n). From that, we can see that the position of the peaks in bias voltage (*V*_b_) shifts when the temperature is in the vicinity of *T*_C_. The physical mechanism of the tunneling processes is shown in Figure 6(o), in which elastic and inelastic tunneling between graphene LLs is presented as red and blue arrows, respectively. By using ferromagnets as a tunneling barrier, the main principle of tunnel junction is based on the inelastic magnon emission below *T*_C_. The increasing temperature would make the system dominant by elastic two-magnon and spin-disorder scattering. A voltage bias can enlarge the difference between the elastic and inelastic processes. The strong proximity effect between CrBr_3_ and graphene suggests that the tunnel junction, in the combination of ferromagnetic and conducting materials at the 2D scale, offers a new strategy for magnon emission and spin injection.

## 6. Conclusion and Future Perspective

In recent years, due to their unique physical properties and application potentials in spintronic devices, 2DMMs have received extensive research attentions [81]. Exploring intrinsic 2D magnetism in such systems is of considerable significance, not only for the understanding of low-dimensional magnetic mechanism but also for the development of next-generation spintronic devices at the atomic scale [82]. In this review, we have summarized the structure characterizations, optical and transport properties, and spintronic devices of 2DMMs. Although the research of this field has been growing rapidly in recent years, there still exist many challenges.

Firstly, it is always promising to enrich the family members of 2DMMs. In general, 2DMMs should not only cover van der Waals crystals but also non-van der Waals ones, such as CrSe, Cr_2_S_3_, and Mn_3_N_2_ [83–86]. On the other hand, to the best of our knowledge, 2DMMs with ferromagnetic or antiferromagnetic order have been reported extensively; however, chiral helimagnetic or topological magnetic order in truly 2D materials is almost unexplored. The realization of chiral helimagnetism and topological spin textures at the 2D scale is crucial for exploring low-dimensional topological materials and quantum information technologies [87]. Thus, more research effort should be devoted to that. Also, it is important to address other issues of 2DMMs by discovering more 2DMM members, such as the ones with high transition temperature, low critical field, and good air stability [88].

Secondly, it is urgent to develop some general, facile, and controllable synthesis methods of 2DMMs according to different needs. At present, the most conventional method for preparing 2DMMs is mechanical exfoliation of bulk crystals. Although this method yields high-quality and ultrathin crystals that are favorable for fundamental studies, we cannot neglect the shortcomings, such as low production, random distribution, and tiny sizes (1-50 *μ*m in general). More synthesis methods, for instance, MBE, chemical vapor deposition (CVD), physical vapor deposition (PVD), and solution-processed methods [89], need to be invented to produce 2DMMs. For instance, O'Hara et al. have succeeded in the growth of a MnSe_x_ monolayer by MBE [90]. The strong ferromagnetism is observed in an epitaxial MnSe_x_ monolayer which originates from a vdW MnSe_2_ monolayer, while the MnSe_x_ multilayer needs to be coalesced with interfacial magnetism of *α*-MnSe(111) to exhibit ferromagnetism. Similarly, a VSe_2_ monolayer has been successfully grown by MBE on van der Waals substrates such as highly oriented pyrolytic graphite (HOPG) and MoS_2_ [91, 92]. Importantly, the VSe_2_ monolayer can persist robust ferromagnetic behavior with a large magnetic moment above room temperature. More recently, electrochemical exfoliation has also been proven to be a promising method to obtain very thin large-sized VSe_2_ flakes with high yields, suggesting that VSe_2_ monolayer flakes are more easily obtained by a electrochemical exfoliation method compared to micromechanical exfoliation [93]. Interestingly, the introduced thiol molecules passivate these VSe_2_ monolayers in the solution growth process and thus well address the instability problem of the VSe_2_ monolayer in ambient. In general, we can gain inspiration from the abundant experience of the synthesis of 2D nonmagnetic materials and conventional magnetic materials to extend these ideas to the newborn 2DMMs. It is not easy but surely possible to fabricate wafer-scale 2DMMs of high quality for practical applications in the near future.

Lastly, the electric-field control on the charge carriers and spins needs to be optimized for spintronic applications of 2DMMs [94]. To achieve this goal, several aspects need to be considered. (i) We should make good use of 2D magnetic heterostructures that can be designed freely and constructed artificially [95]. The atomically sharp interfaces of various 2DMM-based heterostructures are ideal for the spin injection and tunneling at the junction. However, the high-quality interfaces require for careful selection of material components and delicate processing of heterostructure assembly [96]. Therefore, it is desirable to understand the properties of various 2DMMs and to develop efficient technologies for preparing 2D heterostructures. To our delight, both theoretical and experimental investigations can assist a lot in the fabrication of ideal 2DMM heterostructures with designed band alignment and spin flow. (ii) The atomic thickness of 2DMMs should be well utilized, because this structural advantage enables the magnetic properties to be fully modulated by multiple physical fields and their couplings, such as optical, electrical, thermal, and mechanical stress fields [97]. The interplay of orbital character, crystal structure, charge, spin, and exciton would surely expedite the emergence of novel physical phenomena and device tunability of 2DMMs. (iii) It is highly anticipated to combine the merits of 2DMMs with conventional magnetic materials. We can apply a lot of good ideas—some of which are originally from conventional magnetic materials—to 2DMMs. For instance, 2DMMs can also be used to construct nanoscale sensors, magnetic memory, and logic devices. A good example is a highly sensitive magnetic sensor based on a magnetic microwire coil, where monolayer VSe_2_ is working as the magnetic core [98]. The working principle of this sensor is to detect the signal of the resonant frequency of the LC circuit under the varying DC magnetic fields. Furthermore, we can design heterostructures composed of conventional magnetic materials and 2DMMs to explore their interaction effect. The instability in air and incompatibility with solution-processed technology of present 2DMMs might be solved through the media of conventional magnetic materials. Such heterostructures and devices have been successfully fabricated and exhibited good performances [99], yet many other possibilities are eagerly awaited to be created and confirmed in the future. We envision that 2DMMs would help to create a revolutionary development for the highly integrated electronic and spintronic devices in the fields of information computation, communication, and storage technologies.

## References

[1] Griffiths R. B. (1964). Peierls proof of spontaneous magnetization in a two-dimensional Ising ferromagnet. *Physical Review*.

[2] Mermin N. D., Wagner H. (1966). Absence of ferromagnetism or antiferromagnetism in one- or two-dimensional isotropic Heisenberg models. *Physical Review Letters*.

[3] de Jongh L. J., Miedema A. R. (2010). Experiments on simple magnetic model systems. *Advances in Physics*.

[4] Cortie D. L., Causer G. L., Rule K. C., Fritzsche H., Kreuzpaintner W., Klose F. (2020). Two-dimensional magnets: forgotten history and recent progress towards spintronic applications. *Advanced Functional Materials*.

[5] Makarova T. (2009). Nanomagnetism in otherwise nonmagnetic materials. https://arxiv.org/pdf/0904.1550.pdf.

[6] Gutfleisch O., Willard M. A., Brück E., Chen C. H., Sankar S. G., Liu J. P. (2011). Magnetic materials and devices for the 21st century: stronger, lighter, and more energy efficient. *Advanced Materials*.

[7] Brinkman A., Huijben M., van Zalk M. (2007). Magnetic effects at the interface between non-magnetic oxides. *Nature Materials*.

[8] Novoselov K. S., Geim A. K., Morozov S. V. (2004). Electric field effect in atomically thin carbon films. *Science*.

[9] Berger C., Song Z., Li T. (2004). Ultrathin epitaxial graphite: 2D electron gas properties and a route toward graphene-based nanoelectronics. *Journal of Physical Chemistry B*.

[10] Novoselov K. S., Geim A. K., Morozov S. V. (2005). Two-dimensional gas of massless Dirac fermions in graphene. *Nature*.

[11] Bolotin K. I., Sikes K. J., Jiang Z. (2008). Ultrahigh electron mobility in suspended graphene. *Solid State Communications*.

[12] Chen X., Zhou Z., Deng B. (2019). Electrically tunable physical properties of two-dimensional materials. *Nano Today*.

[13] Gong C., Li L., Li Z. (2017). Discovery of intrinsic ferromagnetism in two-dimensional van der Waals crystals. *Nature*.

[14] Huang B., Clark G., Navarro-Moratalla E. (2017). Layer-dependent ferromagnetism in a van der Waals crystal down to the monolayer limit. *Nature*.

[15] Gruber A., Dräbenstedt A., Tietz C., Fleury L., Wrachtrup J., von Borczyskowski C. (1997). Scanning confocal optical microscopy and magnetic resonance on single defect centers. *Science*.

[16] Thiel L., Wang Z., Tschudin M. A. (2019). Probing magnetism in 2D materials at the nanoscale with single-spin microscopy. *Science*.

[17] Li Q., Yang M., Gong C. (2018). Patterning-induced ferromagnetism of Fe_3_GeTe_2_ van der Waals materials beyond room temperature. *Nano Letters*.

[18] Kittel C. (1949). Physical theory of ferromagnetic domains. *Reviews of Modern Physics*.

[19] Yu X. Z., Kanazawa N., Onose Y. (2011). Near room-temperature formation of a skyrmion crystal in thin-films of the helimagnet FeGe. *Nature Materials*.

[20] Yu X. Z., Onose Y., Kanazawa N. (2010). Real-space observation of a two-dimensional skyrmion crystal. *Nature*.

[21] Ding B., Li Z., Xu G. (2019). Observation of magnetic skyrmion bubbles in a van der Waals ferromagnet Fe_3_GeTe_2_. *Nano Letters*.

[22] Togawa Y., Koyama T., Takayanagi K. (2012). Chiral magnetic soliton lattice on a chiral helimagnet. *Physical Review B*.

[23] Wang L., Chepiga N., Ki D. K. (2017). Controlling the topological sector of magnetic solitons in exfoliatedCr1/3NbS2 crystals. *Physical Review Letters*.

[24] Jeong M. S., Namkoong G., Byeon C. C., Kim J. S., Lee H. S. (2014). Optical characterization of nanomaterials. *Journal of Nanomaterials*.

[25] Liu Y., Wang W., Lu H. (2020). The environmental stability characterization of exfoliated few-layer CrXTe_3_(X = Si, Ge) nanosheets. *Applied Surface Science*.

[26] Tian Y., Gray M. J., Ji H., Cava R. J., Burch K. S. (2016). Magneto-elastic coupling in a potential ferromagnetic 2D atomic crystal. *2D Materials*.

[27] Souliou S.-M., Chaloupka J., Khaliullin G. (2017). Raman scattering from Higgs mode oscillations in the two-dimensional antiferromagnet Ca_2_RuO_4_. *Physical Review Letters*.

[28] Lu H., Wang W., Liu Y. (2020). Exfoliation, lattice vibration and air stability characterization of antiferromagnetic van der Waals NiPS_3_ nanosheets. *Applied Surface Science*.

[29] Kim K., Lim S. Y., Lee J. U. (2019). Suppression of magnetic ordering in XXZ-type antiferromagnetic monolayer NiPS_3_. *Nature Communications*.

[30] Xie Q. Y., Wu M., Chen L. M. (2019). Crystallographic and magnetic properties of van der Waals layered FePS_3_ crystal. *Chinese Physics B*.

[31] Long G., Zhang T., Cai X. (2017). Isolation and characterization of few-layer manganese thiophosphite. *ACS Nano*.

[32] Klein D. R., MacNeill D., Song Q. (2019). Enhancement of interlayer exchange in an ultrathin two-dimensional magnet. *Nature Physics*.

[33] Seyler K. L., Zhong D., Klein D. R. (2018). Ligand-field helical luminescence in a 2D ferromagnetic insulator. *Nature Physics*.

[34] Sivadas N., Okamoto S., Xiao D. (2016). Gate-controllable magneto-optic Kerr effect in layered collinear antiferromagnets. *Physical Review Letters*.

[35] Ghazaryan D., Greenaway M. T., Wang Z. (2018). Magnon-assisted tunnelling in van der Waals heterostructures based on CrBr_3_. *Nature Electronics*.

[36] Xing W., Qiu L., Wang X. (2019). Magnon transport in quasi-two-dimensional van der Waals antiferromagnets. *Physical Review X*.

[37] Yuan K., Yao X., Wang H. (2019). Peeling off nanometer-thick ferromagnetic layers and their van der Waals heterostructures. *Advanced Electronic Materials*.

[38] Wang Z., Zhang T., Ding M. (2018). Electric-field control of magnetism in a few-layered van der Waals ferromagnetic semiconductor. *Nature Nanotechnology*.

[39] Wang Z., Gutiérrez-Lezama I., Ubrig N. (2018). Very large tunneling magnetoresistance in layered magnetic semiconductor CrI_3_. *Nature Communications*.

[40] Lin M. W., Zhuang H. L., Yan J. (2016). Ultrathin nanosheets of CrSiTe_3_: a semiconducting two-dimensional ferromagnetic material. *Journal of Materials Chemistry C*.

[41] Jenjeti R. N., Kumar R., Austeria M. P., Sampath S. (2018). Field effect transistor based on layered NiPS_3_. *Scientific Reports*.

[42] Nagaosa N., Sinova J., Onoda S., MacDonald A. H., Ong N. P. (2010). Anomalous Hall effect. *Reviews of Modern Physics*.

[43] Yan J., Luo X., Chen F. C. (2017). Anomalous Hall effect in two-dimensional non-collinear antiferromagnetic semiconductor Cr_0.68_Se. *Applied Physics Letters*.

[44] Wang Q., Sun S., Zhang X., Pang F., Lei H. (2016). Anomalous Hall effect in a ferromagnetic Fe_3_Sn_2_ single crystal with a geometrically frustrated Fe bilayer kagome lattice. *Physical Review B*.

[45] Wang Y., Xian C., Wang J. (2017). Anisotropic anomalous Hall effect in triangular itinerant ferromagnet Fe_3_GeTe_2_. *Physical Review B*.

[46] Fei Z., Huang B., Malinowski P. (2018). Two-dimensional itinerant ferromagnetism in atomically thin Fe_3_GeTe_2_. *Nature Materials*.

[47] Tan C., Lee J., Jung S. G. (2018). Hard magnetic properties in nanoflake van der Waals Fe_3_GeTe_2_. *Nature Communications*.

[48] Liu E., Sun Y., Kumar N. (2018). Giant anomalous Hall effect in a ferromagnetic kagome-lattice semimetal. *Nature Physics*.

[49] Yu R., Zhang W., Zhang H. J., Zhang S. C., Dai X., Fang Z. (2010). Quantized anomalous Hall effect in magnetic topological insulators. *Science*.

[50] Oh S. (2013). The complete quantum Hall trio. *Science*.

[51] Chang C. Z., Zhang J., Feng X. (2013). Experimental observation of the quantum anomalous Hall effect in a magnetic topological insulator. *Science*.

[52] Otrokov M. M., Klimovskikh I. I., Bentmann H. (2019). Prediction and observation of an antiferromagnetic topological insulator. *Nature*.

[53] Deng Y., Yu Y., Shi M. Z. (2020). Quantum anomalous Hall effect in intrinsic magnetic topological insulator MnBi_2_Te_4_. *Science*.

[54] Li J., Li Y., Du S. (2019). Intrinsic magnetic topological insulators in van der Waals layered MnBi_2_Te_4_-family materials. *Science Advances*.

[55] Deng Y., Yu Y., Shi M. Z., Wang J., Chen X. H., Zhang Y. (2019). Magnetic-field-induced quantized anomalous Hall effect in intrinsic magnetic topological insulator MnBi_2_Te_4_. https://arxiv.org/pdf/1904.11468.pdf.

[56] Hu C., Gordon K. N., Liu P. (2020). A van der Waals antiferromagnetic topological insulator with weak interlayer magnetic coupling. *Nature Communications*.

[57] Sun H., Xia B., Chen Z. (2019). Rational design principles of the quantum anomalous Hall effect in superlatticelike magnetic topological insulators. *Physical Review Letters*.

[58] Hu C., Ding L., Gordon K. N. (2019). Realization of an intrinsic, ferromagnetic axion insulator in MnBi_8_Te_13_. https://arxiv.org/pdf/1910.12847.pdf.

[59] Tian S., Gao S., Nie S. (2019). Magnetic topological insulator in MnBi_6_Te_10_ with zero-field ferromagnetic state. https://arxiv.org/pdf/1910.10101.pdf.

[60] Wang Z., Sapkota D., Taniguchi T., Watanabe K., Mandrus D., Morpurgo A. F. (2018). Tunneling spin valves based on Fe_3_GeTe_2_/hBN/Fe_3_GeTe_2_ van der Waals heterostructures. *Nano Letters*.

[61] Ashton M., Gluhovic D., Sinnott S. B., Guo J., Stewart D. A., Hennig R. G. (2017). Two-dimensional intrinsic half-metals with large spin gaps. *Nano Letters*.

[62] Johansen Ø., Risinggård V., Sudbø A., Linder J., Brataas A. (2019). Current control of magnetism in two-dimensionalFe_3_GeTe_2_. *Physical Review Letters*.

[63] Jiang M., Asahara H., Sato S. (2019). Efficient full spin-orbit torque switching in a single layer of a perpendicularly magnetized single-crystalline ferromagnet. *Nature Communications*.

[64] Shao Q., Yu G., Lan Y. W. (2016). Strong Rashba-Edelstein effect-induced spin-orbit torques in monolayer transition metal dichalcogenide/ferromagnet bilayers. *Nano Letters*.

[65] MacNeill D., Stiehl G. M., Guimaraes M. H. D., Buhrman R. A., Park J., Ralph D. C. (2017). Control of spin-orbit torques through crystal symmetry in WTe_2_/ferromagnet bilayers. *Nature Physics*.

[66] Ostwal V., Shen T., Appenzeller J. (2019). Efficient spin-orbit torque switching of the semiconducting van der Waals ferromagnet Cr_2_Ge_2_Te_6_. *Advanced Materials*.

[67] Gong S. J., Gong C., Sun Y. Y. (2018). Electrically induced 2D half-metallic antiferromagnets and spin field effect transistors. *Proceedings of the National Academy of Sciences*.

[68] Jiang S., Li L., Wang Z., Shan J., Mak K. F. (2019). Spin tunnel field-effect transistors based on two-dimensional van der Waals heterostructures. *Nature Electronics*.

[69] Lee S., Choi K. Y., Lee S., Park B. H., Park J. G. (2016). Tunneling transport of mono- and few-layers magnetic van der Waals MnPS_3_. *APL Materials*.

[70] Luong D. H., Phan T. L., Ghimire G., Duong D. L., Lee Y. H. (2019). Revealing antiferromagnetic transition of van der Waals MnPS_3_ via vertical tunneling electrical resistance measurement. *APL Materials*.

[71] Jungwrith T., Marti X., Wadley P., Wunderlich J. (2016). Antiferromagnetic spintronics. *Nature Nanotechnology*.

[72] Wadley P., Howells B., elezny J. (2016). Electrical switching of an antiferromagnet. *Science*.

[73] Kim H. H., Yang B., Tian S. (2019). Tailored tunnel magnetoresistance response in three ultrathin chromium trihalides. *Nano Letters*.

[74] Song T., Cai X., Tu M. W. Y. (2018). Giant tunneling magnetoresistance in spin-filter van der Waals heterostructures. *Science*.

[75] Kim H. H., Yang B., Patel T. (2018). One million percent tunnel magnetoresistance in a magnetic van der Waals heterostructure. *Nano Letters*.

[76] Klein D. R., MacNeill D., Lado J. L. (2018). Probing magnetism in 2D van der Waals crystalline insulators via electron tunneling. *Science*.

[77] Song T., Tu M. W. Y., Carnahan C. (2019). Voltage control of a van der Waals spin-filter magnetic tunnel junction. *Nano Letters*.

[78] Kim H. H., Yang B., Li S. (2019). Evolution of interlayer and intralayer magnetism in three atomically thin chromium trihalides. *Proceedings of the National Academy of Sciences*.

[79] Cai X., Song T., Wilson N. P. (2019). Atomically thin CrCl_3_: an in-plane layered antiferromagnetic insulator. *Nano Letters*.

[80] Wang Z., Gibertini M., Dumcenco D. (2019). Determining the phase diagram of atomically thin layered antiferromagnet CrCl_3_. *Nature Nanotechnology*.

[81] Burch K. S., Mandrus D., Park J. G. (2018). Magnetism in two-dimensional van der Waals materials. *Nature*.

[82] Li H., Ruan S., Zeng Y. J. (2019). Intrinsic van der Waals magnetic materials from bulk to the 2D limit: new frontiers of spintronics. *Advanced Materials*.

[83] Du Z., Yang S., Li S. (2020). Conversion of non-van der Waals solids to 2D transition-metal chalcogenides. *Nature*.

[84] Zhang Y., Chu J., Yin L. (2019). Ultrathin magnetic 2D single-crystal CrSe. *Advanced Materials*.

[85] Zhou S., Wang R., Han J. (2019). Ultrathin non-van der Waals magnetic rhombohedral Cr_2_S_3_: space-confined chemical vapor deposition synthesis and Raman scattering investigation. *Advanced Functional Materials*.

[86] Xiao X., Urbankowski P., Hantanasirisakul K. (2019). Scalable synthesis of ultrathin Mn_3_N_2_ exhibiting room-temperature antiferromagnetism. *Advanced Functional Materials*.

[87] Tokura Y., Yasuda K., Tsukazaki A. (2019). Magnetic topological insulators. *Nature Reviews Physics*.

[88] Kovalev A. A., Sandhoefner S. (2018). Skyrmions and antiskyrmions in quasi-two-dimensional magnets. *Frontiers in Physics*.

[89] May A. F., Ovchinnikov D., Zheng Q. (2019). Ferromagnetism near room temperature in the cleavable van der Waals crystal Fe_5_GeTe_2_. *ACS Nano*.

[90] O’Hara D. J., Zhu T., Trout A. H. (2018). Room temperature intrinsic ferromagnetism in epitaxial manganese selenide films in the monolayer limit. *Nano Letters*.

[91] Coelho P. M., Nguyen Cong K., Bonilla M. (2019). Charge density wave state suppresses ferromagnetic ordering in VSe_2_ monolayers. *The Journal of Physical Chemistry C*.

[92] Bonilla M., Kolekar S., Ma Y. (2018). Strong room-temperature ferromagnetism in VSe_2_ monolayers on van der Waals substrates. *Nature Nanotechnology*.

[93] Yu W., Li J., Herng T. S. (2019). Chemically exfoliated VSe_2_ monolayers with room-temperature ferromagnetism. *Advanced Materials*.

[94] Železný J., Wadley P., Olejník K., Hoffmann A., Ohno H. (2019). Publisher correction: spin transport and spin torque in antiferromagnetic devices. *Nature Physics*.

[95] Zhang W., Wong P. K. J., Zhu R., Wee A. T. S. (2019). van der Waals magnets: wonder building blocks for two-dimensional spintronics?. *InfoMat*.

[96] Gibertini M., Koperski M., Morpurgo A. F., Novoselov K. S. (2019). Magnetic 2D materials and heterostructures. *Nature Nanotechnology*.

[97] Gong C., Zhang X. (2019). Two-dimensional magnetic crystals and emergent heterostructure devices. *Science*.

[98] Jimenez V. O., Kalappattil V., Eggers T. (2020). A magnetic sensor using a 2D van der Waals ferromagnetic material. *Scientific Reports*.

[99] Matsukura F., Tokura Y., Ohno H. (2015). Control of magnetism by electric fields. *Nature Nanotechnology*.

